# A novel method to increase transgene expression and the stability of gene therapy-associated episomal vectors: A novel method to increase transgene expression and stability

**DOI:** 10.3724/abbs.2025104

**Published:** 2025-07-23

**Authors:** Xi Zhang, Rui Liu, Zimeng Han, Zihan Guo, Mengying Ji, Wen Wang, Yanlong Jia, Tianyun Wang, Xiaoyin Wang

**Affiliations:** 1 School of Pharmacy Henan Medical University Xinxiang 453003 China; 2 School of Life Science and Technology North Henan Medical University Xinxiang 453003 China; 3 School of Basic Medical Sciences Henan Medical University Xinxiang 453003 China; 4 International Joint Research Laboratory for Recombinant Pharmaceutical Protein Expression System of Henan Henan Medical University Xinxiang 453003 China

Non-viral episomal vectors offer a safe and attractive alternative to viral and integrated vectors by avoiding insertional mutagenesis and position effects, making them ideal expression vectors for gene therapy [1]. The first non-viral episomal vector, pEPI-1, which is based on the full-length scaffold/matrix attachment region (S/MAR), was established by Piechaczek *et al*. [2]. The full-length S/MAR element interacts with the nuclear matrix via the matrix protein, *e.g.* SAF-A, thereby maintaining mitotic stability and transgene expression [ 3, 4] . Several strategies, including optimization of the vector backbone and promoter and incorporation of chromatin-modifying elements, have been used to increase expression levels and stability [ 5– 8] . In our previous work, we constructed the novel vector pEGFP-C1-M on the basis of S/MAR characteristic motifs (only 375 bp) [9]. This vector, which is shorter than the prototype episomal vector pEPI-1, resulted in relatively higher transgene expression. Building on the pEGFP-C1-M vector, we further constructed the episomal vector pEMEα with the EF-1α promoter and demonstrated that pEMEα maintained higher transgene expression, stability and copy number [10].

The transgene expression levels of episomal vectors are correlated with gene copy number, that is, the number of plasmid episomes on the host cell chromosome. Previous studies have shown that the episomal maintenance of pEPI-1 vectors is mediated primarily by SAF-A [2]. While the role of SAF-A in maintaining mammalian pEPI-1 episomal vectors has been well established [ 3, 4] , it remains unknown whether the overexpression of SAF-A promotes transgene expression and stability and whether the 375 bp MAR characteristic sequence retains its interaction with SAF-A.

In the present study, we first evaluated whether transgene expression is positively correlated with the expression level of SAF-A. The non-viral episomal vector pEMEα was used as the gene of interest (GOI) vector ( Figure 1A) and was subsequently transfected into CHO-K1 cells using the Lipofectamine 2000 reagent (Invitrogen, Carlsbad, USA). The cells were cultured in medium containing 800 μg/mL geneticin (G418; Beyotime, Shanghai, China) 48 h post-transfection, and the G418 concentration was then reduced to 400 μg/mL to obtain monoclonal cell lines using the limiting dilution method. Five monoclonal cell clones were selected, and the eGFP expression levels, measured as the mean fluorescence intensity (MFI), were (6.5 ± 1.0) × 10 ^4^, (6.8 ± 0.9) × 10 ^4^, (7.0 ± 1.4) × 10 ^4^, (23.5 ± 1.2) × 10 ^4^ and (14.9 ± 0.17) × 10 ^4^ for Clones 1–5, respectively ( Figure 1B). qPCR analysis of Clones 1–5 revealed that the relative mRNA levels of *SAF-A* and *eGFP* were 0.16 ± 0.13, 0.43 ± 0.11, 0.46 ± 0.23, 2.17 ± 0.41, 1.78 ± 0.15 and 0.51 ± 0.16, 0.71 ± 0.12, 1.05 ± 0.09, 2.47 ± 0.14, and 1.86 ± 0.10, respectively ( Figure 1C). Our results indicated that eGFP mRNA and protein expression levels are positively correlated with *SAF-A* mRNA level ( Figure 1D).

**Figure 1:**
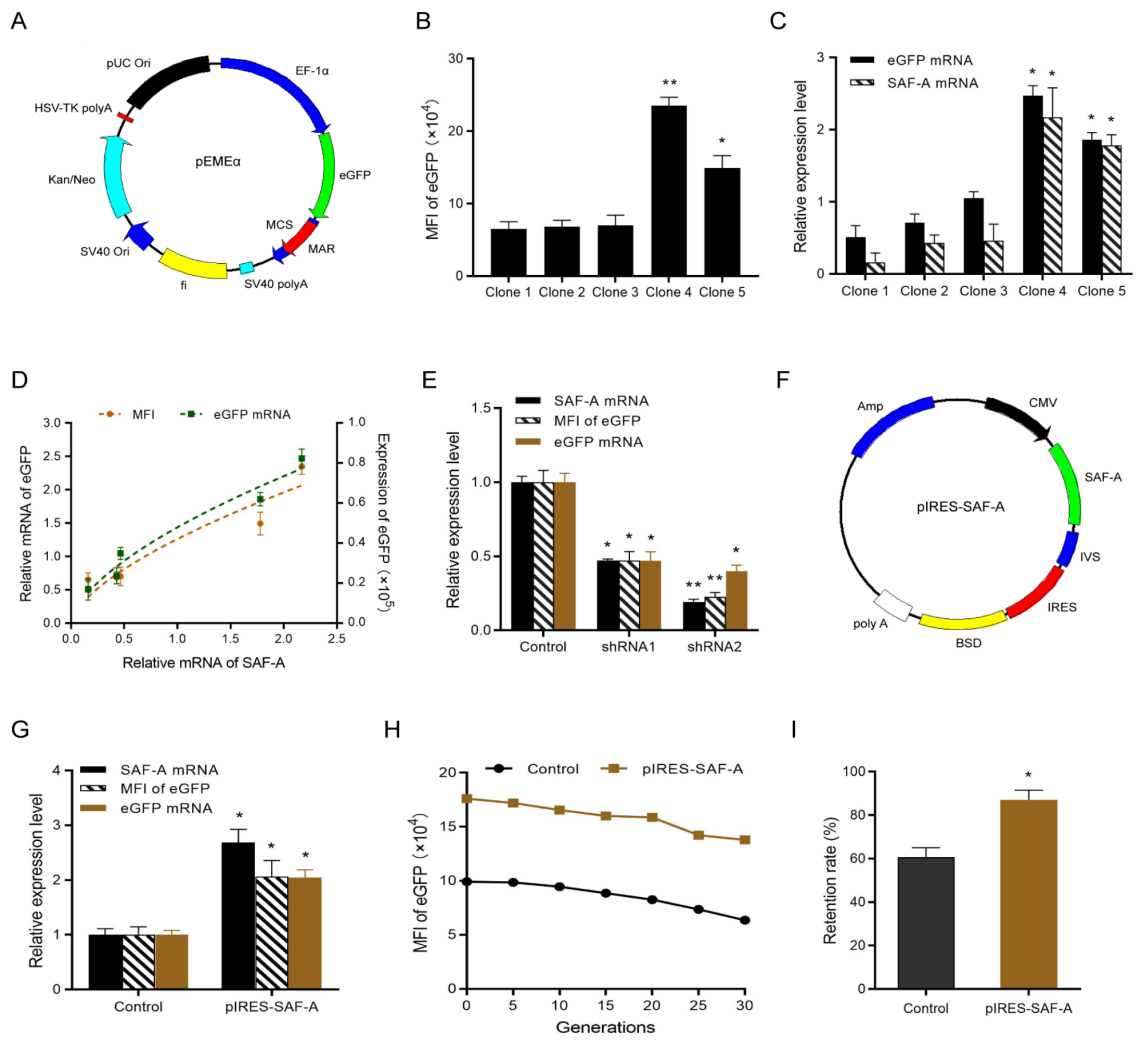
Figure 1 Relationship between SAF-A expression levels and transgene expression and stability (A) Map of the optimized non-viral episomal vector pEMEα. (B) MFI of five monoclonal cell clones stably transfected with the pEMEα vector. Compared with Clone 1, *P < 0.05, **P < 0.01. (C) Relative mRNA levels of eGFP and SAF-A in Clones 1–5. Compared with Clone 1, *P < 0.05. (D) Correlation between SAF-A mRNA levels and eGFP mRNA and protein expression levels. (E) qPCR and flow cytometry were used to measure SAF-A and eGFP mRNA and protein expression levels, respectively. Compared with the control, *P < 0.05, **P < 0.01. (F) Map of the pIRES-SAF-A vector. (G) Relative expression levels of SAF-A mRNA and eGFP in pools of cells stably transfected with the pIRES-SAF-A vector. Compared with the control, *P < 0.05. (H) Long-term transgene expression stability was assessed at 0, 5, 10, 15, 20, 25, and 30 generations. (I) The retention rate of transgene expression was calculated before and after 30 generations. Compared with the control, *P < 0.05.

To further verify the relationship between SAF-A expression and transgene expression, two shRNA plasmids targeting SAF-A (shRNA1: 5′-GCCACCTGTTGAAGAAGAAGA-3′, and shRNA2: 5′-GCTGGAGGAAGAGCTTCTTAT-3′) which were obtained from Shanghai GenePharma Co., Ltd. (Shanghai, China) were designed and transfected into stable cell pools with the pEMEα vector. qPCR analysis revealed that the relative *SAF-A* mRNA levels in the shRNA1 and shRNA2 vectors were 0.47 ± 0.01 and 0.19 ± 0.02, respectively, indicating successful downregulation of SAF-A expression ( Figure 1E). Moreover, flow cytometry and qPCR revealed that, compared with those in the control group, the relative protein and mRNA levels of eGFP were reduced by 0.47- and 0.23-fold, and 0.47- and 0.40-fold in the pools of cells transfected with the shRNA1 and shRNA2 vectors, respectively ( Figure 1E).

On the basis of the above results, the SAF-A overexpression vector pIRES-SAF-A ( Figure 1F) was constructed and transfected into CHO-K1 cells, and the cells were cultured in blasticidin-containing medium 48 h after transfection to obtain stable cell pools. The stable cell pools overexpressing SAF-A were subsequently transfected with the pEMEα vector. Stable cell pools coexpressing SAF-A and GOI were selected, and the relative mRNA levels of *SAF-A* and *eGFP* were analyzed. qPCR analysis revealed that the relative mRNA levels of *SAF-A* and *eGFP* in the pools of cells overexpressing SAF-A were 2.69-fold and 2.05-fold higher than those in the control group, respectively ( Figure 1G). Flow cytometry also revealed a 2.07-fold increase in MFI in stable cell pools overexpressing SAF-A compared with the control group ( Figure 1G). To assess the long-term stability of transgene expression, we measured the MFI in stable cell pools overexpressing SAF-A at 0, 5, 10, 15, 20, 25, and 30 generations and calculated the maintenance rate of transgene expression by comparing MFI values before and after 30 generations. The results revealed that the eGFP expression levels gradually decreased over time. However, eGFP expression in cell pools overexpressing SAF-A remained higher than that in the control group after 30 generations ( Figure 1H). After 30 generations of long-term culture, the SAF-A-overexpressing cell pools retained 87.00% ± 7.94% of the original eGFP expression levels, whereas the control group presented a decrease to 60.66% ± 7.57% ( Figure 1I). These data revealed that overexpression of the helper gene *SAF-A* improved transgene expression and the stability of episomal vectors in CHO cells.

To investigate the mechanism by which SAF-A enhances transgene expression, the gene copy numbers were analyzed. Hirt DNA was extracted from pools of cells overexpressing SAF-A after 30 generations, and a plasmid rescue assay was performed to evaluate the status of vector replication. The results indicated that the plasmid DNA from cell pools overexpressing SAF-A was identical to the original plasmid DNA ( Figure 2A). Moreover, qPCR analysis revealed that the relative GOI copy number in cell pools overexpressing SAF-A (6.15 ± 0.50) was significantly higher than that in the control group (1.63 ± 0.56; Figure 2B). These findings revealed that the episomal status of the vector was maintained after long-term culture and that the overexpression of SAF-A increased the GOI copy number in CHO cells. In addition, we also found that SAF-A could maintain stable expression for 30 generations after transfection with the helper vector pIRES-SAF-A. Thus, we speculated that stable overexpression of SAF-A might lead to more stable episomes on the host cell chromosome, thereby improving transgene expression and stability.

**Figure 2:**
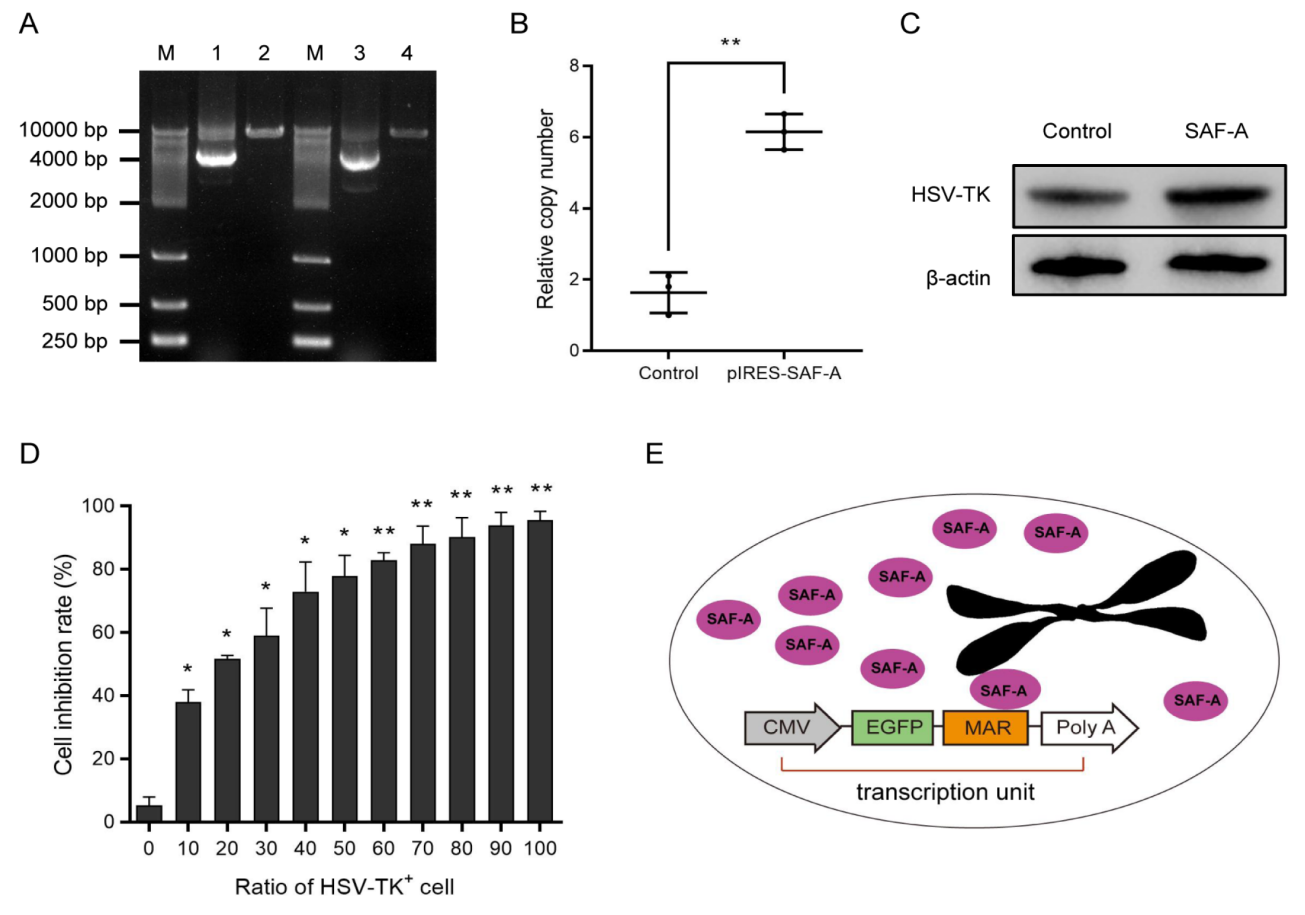
Figure 2 Mechanism of SAF-A enhancing transgene expression mediated by episomal vectors (A) Episomal localization of the vector was determined after 30 generations by plasmid rescue experiments. M, DL10000 marker; Lane 1, pEMEα plasmid; Lane 2, pEMEα plasmid digested with NheI; Lane 3, Hirt DNA isolated from a single bacterial clone; Lane 4, Hirt DNA isolated from a single bacterial clone digested with NheI. (B) Relative GOI copy numbers in cell pools overexpressing SAF-A after 30 generations were assessed by qPCR analysis. Compared with the control, **P < 0.01. (C) The effect of SAF-A on HSV-TK expression was measured by western blot analysis. (D) A bystander effect assay was performed to detect the function of HSV-TK in HCT116 cells. Compared with the HSV-TK+ ratio of 0%, *P < 0.05, **P < 0.01. (E) Proposed mechanism of episomal replication by S/MAR binding to the nuclear matrix protein SAF-A.

Moreover, the eGFP reporter gene was replaced by the *HSV-TK* gene (a suicide gene) to analyze the effects of SAF-A on transgene expression. The pIRES-SAF-A vector was transfected into HCT116 cells, and the cells were cultured in medium containing blasticidin 48 h after transfection. Then, stable cell pools overexpressing SAF-A were transfected with the pEME-HSV-TK plasmid and screened. Western blot analysis results demonstrated that the expression of HSV-TK in stably cotransfected cells was higher than that in the control group (pEME-HSV-TK plasmid alone) ( Figure 2C). A bystander effect assay was used to assess the function of HSV-TK. Stably transfected HCT116 cells with the pEME-HSV-TK vector were mixed with untransfected HCT116 cells at different ratios, and then, ganciclovir (GCV) was added to the medium at a final concentration of 80 μg/mL. The results revealed that a small number of stably transfected HCT116 cells could induce the apoptosis of most cells, indicating that the expressed suicide gene *HSV-TK* was functional ( Figure 2D).

In conclusion, we demonstrated for the first time that *SAF-A* acts as a helper gene to improve transgene expression and stability mediated by episomal vectors in CHO cells. A transcription unit includes GOI and S/MAR, and S/MAR sequences can mediate the binding of early replication sites to SAF-A to stabilize the episomal presence ( Figure 2E). These findings support the safe and long-term stable expression of GOIs, which provides a novel and efficient method for improving transgene expression and stability mediated by episomal vectors for gene therapy.

## References

[1] Sayed N, Allawadhi P, Khurana A, Singh V, Navik U, Pasumarthi SK, Khurana I (2022). Gene therapy: comprehensive overview and therapeutic applications. Life Sci.

[2] Piechaczek C, Fetzer C, Baiker A, Bode J, Lipps HJ (1999). A vector based on the SV40 origin of replication and chromosomal S/MARs replicates episomally in CHO cells. Nucleic Acids Res.

[3] Schaarschmidt D, Baltin J, Stehle IM, Lipps HJ, Knippers R (2004). An episomal mammalian replicon: sequence-independent binding of the origin recognition complex. EMBO J.

[4] Jenke ACW, Stehle IM, Herrmann F, Eisenberger T, Baiker A, Bode J, Fackelmayer FO (2004). Nuclear scaffold/matrix attached region modules linked to a transcription unit are sufficient for replication and maintenance of a mammalian episome. Proc Natl Acad Sci USA.

[5] Haase R, Argyros O, Wong SP, Harbottle RP, Lipps HJ, Ogris M, Magnusson T (2010). pEPito: a significantly improved non-viral episomal expression vector for mammalian cells. BMC Biotechnol.

[6] Argyros O, Wong SP, Fedonidis C, Tolmachov O, Waddington SN, Howe SJ, Niceta M (2011). Development of S/MAR minicircles for enhanced and persistent transgene expression in the mouse liver. J Mol Med.

[7] Stavrou EF, Lazaris VM, Giannakopoulos A, Papapetrou E, Spyridonidis A, Zoumbos NC, Gkountis A (2017). The β-globin replicator greatly enhances the potential of S/MAR based episomal vectors for gene transfer into human haematopoietic progenitor cells. Sci Rep.

[8] Hagedorn C, Antoniou MN, Lipps HJ (2013). Genomic
*cis*-acting sequences improve expression and establishment of a nonviral vector. Mol Ther Nucleic Acids.

[9] Lin Y, Li Z, Wang T, Wang X, Wang L, Dong W, Jing C (2015). MAR characteristic motifs mediate episomal vector in CHO cells. Gene.

[10] Wang X, Xu Z, Tian Z, Zhang X, Xu D, Li Q, Zhang J (2017). The EF-1α promoter maintains high-level transgene expression from episomal vectors in transfected CHO-K1 cells. J Cell Mol Med.

